# Supplementary material for: Morphometric Study of the Initial Ventricular Indices to Predict the Complications and Outcome of Aneurysmal Subarachnoid Hemorrhage
Source: J Clin Med. 2023 Mar 29;12(7):2585. doi: 10.3390/jcm12072585 (PMC10095006; doi:10.3390/jcm12072585)
Supplement: Supplementary file 1 [file jcm-12-02585-s001.zip › jcm-2101374-supplementary.pdf]

Journal of
Clinical Medicine

SUPPLEMENTARY MATERIALS

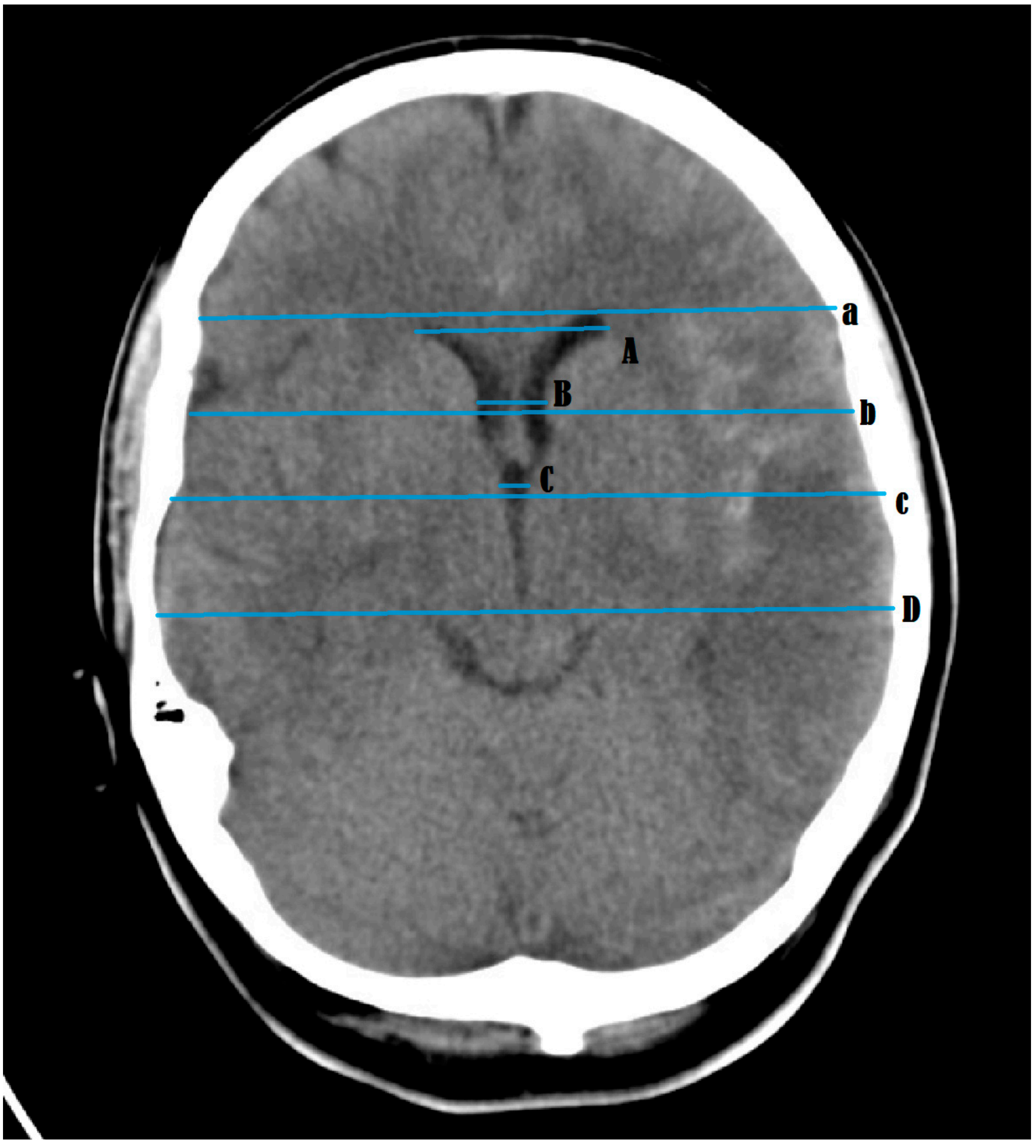

Figure S1. Illustration of the ventricular measurements. **Bifrontal ratio**: maximum width between the two frontal horns (A)/internal width of the vault at same level (a). **Bicaudate ratio**: minimum width of the ventricles between caudate nuclei (B)/internal width of the vault at same level (b). **Ventricular ratio**: minimum width of the ventricles (B)/maximum width between frontal horns (A). **Third ventricle ratio**: greatest width of the third ventricle (C)/internal width of the vault at same level (c). **Evans' index**: maximum width between frontal horns (A)/maximum internal width of the vault (D). **Huckman's index**: maximum width between the two frontal horns (A) + minimum width of the ventricles between caudate nuclei (B).

Table S1. Exploratory analyses based on the bivariate associations between the ventricular indices (as continuous variables) and other parameters included in this study.

Copyright: © 2023 by the authors. Licensee MDPI, Basel, Switzerland. This article is an open access article distributed under the terms and conditions of the Creative Commons Attribution (CC BY) license (https://creativecommons.org/licenses/by/4.0/).

Parameter	Stats	Bifrontal ratio	Bicaudate ratio	Evans' index	Ventricular ratio	Huckmans' index	3rd ventricle ratio
Age, per year increase	CC	0.275	0.370	0.203	0.358	0.318	0.375
	P-value	**<0.0001**	**<0.0001**	**<0.0001**	**<0.0001**	**<0.0001**	**<0.0001**
Female sex	CC	−0.048	−0.052	−0.082	−0.061	−0.158	−0.050
	P-value	0.200	0.163	**0.027**	**0.100**	**<0.0001**	0.181
WFNS grade 4–5	CC	0.124	0.116	0.072	0.100	0.085	0.133
	P-value	**0.001**	**0.002**	0.053	**0.007**	**0.021**	**<0.0001**
Fisher grade 3–4	CC	0.193	0.184	0.144	0.138	0.150	0.182
	P-value	**<0.0001**	**<0.0001**	**<0.0001**	**<0.0001**	**<0.0001**	**<0.0001**
Clipping	CC	−0.213	−0.267	−0.227	−0.245	−0.262	−0.207
	P-value	**<0.0001**	**<0.0001**	**<0.0001**	**<0.0001**	**<0.0001**	**<0.0001**
Increased leukocyte count at admission	CC	0.006	−0.017	0.007	−0.005	−0.007	−0.027
	P-value	0.882	0.680	0.863	0.898	0.870	0.520
Increased CRP at admission	CC	0.098	0.135	0.102	0.126	0.143	0.182
	P-value	**0.019**	**0.001**	**0.014**	**0.002**	**<0.0001**	**<0.0001**
Increased IL-6 in CSF at admission	CC	0.078	0.089	0.083	0.051	0.096	0.072
	P-value	0.148	0.096	0.120	0.342	0.069	0.179
Increased body temperature at admission	CC	−0.045	−0.059	−0.063	−0.040	−0.048	−0.056
	P-value	0.256	0.140	0.108	0.305	0.223	0.158
SEBES 3–4	CC	−0.163	−0.234	−0.178	−0.214	−0.245	−0.265
	P-value	**<0.0001**	**<0.0001**	**<0.0001**	**<0.0001**	**<0.0001**	**<0.0001**
Decompressive craniectomy	CC	−0.184	−0.265	−0.195	−0.265	−0.259	−0.259
	P-value	**<0.0001**	**<0.0001**	**<0.0001**	**<0.0001**	**<0.0001**	**<0.0001**
Occurrence of ICP increase	CC	−0.062	−0.114	−0.070	−0.114	−0.106	−0.161
	P-value	0.103	**0.002**	0.059	**0.002**	**0.004**	**<0.0001**
Duration of ICP increase	CC	−0.075	−0.126	−0.083	−0.135	−0.119	−0.180
	P-value	0.056	**0.001**	**0.033**	**<0.0001**	**0.002**	**<0.0001**
Aneurysm rebleeding	CC	0.019	0.049	0.003	0.054	0.033	0.037
	P-value	0.607	0.196	0.928	0.143	0.376	0.325
DIND	CC	−0.015	0.055	0.005	0.063	0.027	0.063
	P-value	0.721	0.181	0.896	0.121	0.508	0.125
TCD vasospasm	CC	−0.102	−0.108	−0.070	−0.095	−0.112	−0.099
	P-value	**0.012**	**0.008**	**0.079**	**0.017**	**0.005**	**0.014**
Angiographically treated vasospasm	CC	−0.021	−0.008	−0.010	0.014	−0.027	−0.004
	P-value	0.578	0.827	0.795	0.715	0.462	0.915
Early angiographic vasospasm (<72h after ictus)	CC	−0.088	0.064	0.093	0.016	0.071	0.002
	P-value	**0.038**	0.131	**0.026**	0.695	0.086	0.960
Increased duration of mechanical ventilation	CC	0.050	0.075	0.029	0.064	0.041	0.061
	P-value	0.189	**0.049**	0.436	0.087	0.277	0.107
Increased mean 14-days CRP during SAH	CC	0.027	0.039	0.008	0.042	0.016	0.079
	P-value	0.518	0.352	0.850	0.310	0.695	0.060
Increased mean 14-days leukocyte count during SAH	CC	0.041	0.030	0.023	0.014	0.003	0.027
	P-value	0.321	0.477	0.577	0.737	0.941	0.523
Increased mean 14-days IL-6 in CSF	CC	0.187	0.258	0.194	0.226	0.254	0.193
	P-value	**<0.0001**	**<0.0001**	**<0.0001**	**<0.0001**	**<0.0001**	**<0.0001**
Fever duration, per day increase	CC	0.101	0.126	0.087	0.103	0.106	0.087
	P-value	**0.010**	**0.001**	**0.025**	**0.007**	**0.006**	**0.025**
Systemic infection	CC	0.070	0.068	0.044	0.041	0.042	0.011

	P-value	0.077	0.084	0.259	0.299	0.284	0.778
Acute coronary syndrome	CC	0.081	0.079	0.056	0.052	0.061	0.070
	P-value	**0.045**	0.051	0.158	0,192	0.127	0.081
Acute renal failure	CC	0.093	0.106	0.060	0.091	0.077	0.088
	P-value	**0.021**	**0.009**	0.134	**0.022**	0.054	**0.028**
Epilepsy	CC	−0.046	−0.070	−0.033	−0.063	−0.054	−0.047
	P-value	0.221	0.061	0.372	0.089	0.144	0.209
Cerebral infarction	CC	0.068	0.109	0.060	0.119	0.074	0.101
	P-value	0.072	**0.004**	0.105	**0.001**	0.046	**0.007**
In-hospital mortality	CC	0.099	0.113	0.067	0.114	0.088	0.113
	P-value	**0.008**	**0.003**	**0.069**	**0.002**	**0.017**	**0.002**
Unfavorable outcome at 6 months (mRs >3)	CC	0.163	0.190	0.123	0.175	0.160	0.179
	P-value	**<0.0001**	**<0.0001**	**0.001**	**<0.0001**	**<0.0001**	**<0.0001**

Abbreviations: CC – correlation coefficient, DIND= delayed ischemic neurological deficit, SEBES= Subarachnoid Early Brain Edema Score, CRP= C-reactive protein, IL-6= interleukin-6, ICP= intracranial pressure, TCD= transcranial Doppler sonography, CSF= cerebrospinal fluid, WFNS= World Federation of Neurosurgical Societies, mRs= modified Rankin scale. Significant findings are in **bold**.

Table S2. ROC analysis for the association between the ventricular measurements and primary study endpoint (unfavorable outcome at 6 months after SAH defined as modified Rankin scale >3). Based on the AUC values a clinically relevant cut-off was determined for the ventricular ratios and indices.

Ventricular measurements/indices	AUC (95 % CI)	p-value	Selected cut-off
**Bifrontal ratio: A/a**	0.603 (0.559–0.648)	**<0.0001**	0.337
**Bicaudate ratio: B/b**	0.624 (0.579–0.669)	**<0.0001**	0.175
**Ventricular ratio: B/A**	0.614 (0.569–0.660)	**<0.0001**	0.559
**Huckman's index: A+B**	0.618 (0.573–0.663)	**<0.0001**	5.480
**Evans' index: A/D**	0.590 (0.544–0.635)	**<0.0001**	0.281
**Third ventricle ratio: C/c**	0.613 (0.568–0.658)	**<0.0001**	0.053

Abbreviations: ROC= receiver operating characteristics, AUC= area under the curve, CI= confidence interval, A= maximum width between the two frontal horns, a= internal width of the vault at level of A, B= minimum width of the ventricles between caudate nuclei, b= internal width of the vault at level of B, C= greatest width of the third ventricle, c= internal width of the vault at level of C, D= maximum internal width of the vault. Significant values are in **bold.**

Table S3. Univariate analysis of the ventricular indices and other categorical variables. Ventricular indices are dichotomized at clinically relevant cut-off values, according to the AUC of the ROC curves. All major endpoint, as well as older age, clipping, severe SEBES, IVH, DC and infectious parameters are significantly correlated to higher ventricular indices.

	**Bifrontal ratio ≥ 0.337**		**Bicaudate ratio ≥ 0.175**		**Evans' index ≥ 0.281**		**Ventricular ratio ≥ 0.559**		**Huckman's index ≥ 5.48**		**3rd ventricle ratio ≥ 0.053**	
	**OR (95% CI)**	**p-value**	**OR (95% CI)**	**p-value**	**OR (95% CI)**	**p-value**	**OR (95% CI)**	**p-value**	**OR (95% CI)**	**p-value**	**OR (95% CI)**	**p-value**
**Parameter**												
**Patient characteristics**												
Age>55 years	**2.79 (2.05–3.79)**	**<0.0001**	**3.49 (2.55–4.78)**	**<0.0001**	**2.09 (1.55–2.82)**	**<0.0001**	**3.45 (2.50–4.75)**	**<0.0001**	**3.32 (2.45–4.51)**	**<0.0001**	**3.14 (2.30–4.28)**	**<0.0001**
Female sex	0.87 (0.64–1.19)	0.425	0.83 (0.61–1.15)	0.289	0.74 (0.55–1.02)	0.068	0.75 (0.54–1.03)	0.082	**0.51 (0.38–0.70)**	**<0.0001**	0.75 (0.55–1.03)	0.080
**SAH characteristics**												
WFNS 4–5	**1.35 (1.01–1.82)**	**0.05**	**1.84 (1.36–2.49)**	**<0.001**	1.22 (0.91–1.64)	0.202	**1.74 (1.28–2.37)**	**<0.001**	**1.45 (1.08–1.93)**	**0.014**	**1.43 (1.06–1.92)**	**0.02**
Fisher 3–4	**2.48 (1.46–4.20)**	**<0.001**	**2.72 (1.50–4.92)**	**<0.001**	**2.02 (1.18–3.47)**	**0.011**	**1.98 (1.11–3.54)**	**0.024**	**2.19 (1.28–3.74)**	**0.004**	**2.70 (1.60–4.56)**	**<0.001**
SEBES 3–4	**0.55 (0.41–0.74)**	**<0.0001**	**0.48 (0.35–0.65)**	**<0.0001**	**0.55 (0.41–0.74)**	**<0.0001**	**0.57 (0.42–0.78)**	**<0.0001**	**0.44 (0.33–0.60)**	**<0.0001**	**0.50 (0.37–0.63)**	**<0.0001**
IVH	**4.22 (3.08–5.78)**	**<0.0001**	**4.44 (3.19–6.16)**	**<0.0001**	**4.36 (3.17–5.99)**	**<0.0001**	**3.33 (2.40–4.63)**	**<0.0001**	**4.85 (3.54–6.65)**	**<0.0001**	**4.80 (3.49–6.58)**	**<0.0001**
Clipping	**0.49 (0.36–0.67)**	**<0.0001**	**0.40 (0.28–0.55)**	**<0.0001**	**0.51 (0.38–0.70)**	**<0.0001**	**0.52 (0.37–0.73)**	**<0.0001**	**0.42 (0.31–0.57)**	**<0.0001**	**0.52 (0.38–0.70)**	**<0.0001**
**SAH complications**												
Aneurysm rebleeding	0.98 (0.54–1.78)	1.000	1.71 (0.94–3.11)	0.087	0.96 (0.53–1.73)	1.000	**1.89 (1.04–3.42)**	**0.039**	1.08 (0.60–1.94)	0.881	0.99 (0.55–1.80)	1.000
DC	**0.49 (0.35–0.69)**	**<0.0001**	**0.39 (0.27–0.56)**	**<0.0001**	**0.53 (0.38–0.74)**	**<0.0001**	**0.36 (0.24–0.52)**	**<0.0001**	**0.39 (0.28–0.55)**	**<0.0001**	**0.42 (0.30–0.58)**	**<0.0001**
Pathologic ICPs ≥1 day	0.81 (0.60–1.09)	0.174	0.83 (0.61–1.12)	0.219	0.81 (0.61–1.09)	0.176	0.84 (0.62–1.15)	0.307	**0.66 (0.49–0.88)**	**0.006**	**0.64 (0.47–0.86)**	**0.003**
DIND	1.15 (0.81–1.63)	0.475	1.30 (0.92–1.86)	0.146	1.06 (0.75–1.50)	0.790	1.34 (0.94–1.92)	0.116	1.06 (0.75–1.50)	0.792	1.39 (0.98–1.98)	0.075

TCD vasospasms	**0.71 (0.51–0.97)**	**0.035**	**0.61 (0.44–0.85)**	**0.004**	0.82 (0.59–1.12)	0.225	**0.71 (0.51–0.99)**	**0.050**	**0.67 (0.49–0.91)**	**0.013**	0.76 (0.55–1.04)	0.090
Endovascular vasospasm treatment	1.02 (0.72–1.45)	0.929	1.03 (0.72–1.47)	0.928	1.10 (0.77–1.55)	0.657	1.26 (0.88–1.79)	0.230	0.90 (0.64–1.27)	0.599	1.08 (0.76–1.53)	0.723
Early angiographic vasospasm #	1.76 (0.98–3.17)	0.063	1.48 (0.84–2.59)	0.192	1.50 (0.85–2.60)	0.198	1.19 (0.67–2.11)	0.554	1.49 (0.86–2.58)	0.165	0.87 (0.50–1.50)	0.672
Epileptic seizures	0.80 (0.48–1.33)	0.439	0.68 (0.40–1.17)	0.187	0.75 (0.44–1.27)	0.299	0.59 (0.33–1.06)	0.077	−0.74 (0.44–1.24)	0.301	0.71 (0.42–1.17)	0.196
**Other complications**												
Mechanical ventilation ≥7 days	1.07 (0.79–1.44)	0.703	1.24 (0.92–1.69)	0.164	1.03 (0.77–1.39)	0.880	1.12 (0.82–1.53)	0.479	1.08 (0.80–1.45)	0.653	1.15 (0.85–1.54)	0.404
Fever duration, ≥5 days	**1.39 (1.02–1.88)**	**0.042**	**1.44 (1.05–1.97)**	**0.025**	**1.42 (1.05–1.93)**	**0.029**	1.20 (0.87–1.65)	0.291	1.36 (1.00–1.84)	0.054	1.30 (0.96–1.77)	0.102
Systemic infections	1.26 (0.92–1.73)	0.152	**1.40 (1.02–1.92)**	**0.043**	1.23 (0.91–1.69)	0.179	1.27 (0.92–1.75)	0.162	1.18 (0.87–1.60)	0.310	1.02 (0.75–1.39)	0.937
Sepsis	1.39 (0.61–3.18)	0.534	1.54 (0.68–3.49)	0.297	1.25 (0.56–2.78)	0.681	1.78 (0.80–3.97)	0.197	1.27 (0.57–2.81)	0.684	0.68 (0.31–1.53)	0.416
ACS	2.00 (0.74–5.40)	0.231	2.13 (0.84–5.38)	0.151	1.20 (0.48–3.00)	0.815	1.72 (0.69–4.31)	0.327	2.21 (0.87–5.61)	0.111	2.07 (0.78–5.45)	0.172
Acute kidney failure	6.84 (0.84–55.93)	0.069	**10.50 (1.28–85.88)**	**0.009**	1.66 (0.44–6.23)	0.510	3.71 (0.92–14.96)	0.075	4.10 (0.84–19.87)	0.090	6.85 (0.85–55.1)	0.044
**Outcome after SAH**												
Cerebral infarction(s)	**1.36 (1.01–1.83)**	**0.050**	**1.71 (1.26–2.32)**	**0.001**	**1.36 (1.01–1.83)**	**0.043**	**1.81 (1.32–2.47)**	**<0.0001**	**1.36 (1.02–1.82)**	**0.045**	**1.54 (1.15–2.07)**	**0.004**
In–hospital mortality *	**1.69 (1.17–2.44)**	**0.006**	**1.95 (1.35–2.80)**	**<0.0001**	**1.64 (1.15–2.34)**	**0.008**	**1.92 (1.33–2.75)**	**0.001**	**1.64 (1.15–2.34)**	**0.007**	**1.69 (1.17–2.46)**	**0.006**
Unfavorable outcome ^	**1.96 (1.43–2.68)**	**<0.0001**	**2.56 (1.86–3.54)**	**<0.0001**	**1.84 (1.35–2.52)**	**<0.0001**	**2.61 (1.88–3.61)**	**<0.0001**	**2.07 (1.52–2.83)**	**<0.0001**	**1.89 (1.38–2.59)**	**<0.0001**

Abbreviations: OR= odds ratio, SD= standard deviation, CI= confidence interval, AUC= area under the curve, ROC= receiver operating characteristics, SEBES= subarachnoid early brain edema score, DC= decompressive craniectomy, ICP= intracranial pressure, IVH= intraventricular hemorrhage, CRP= C-reactive protein, DIND= delayed ischemic neurological deficit, TCD= transcranial Doppler sonography, VS= vasospasms, ACS= acute coronary syndrome, DCI= delayed cerebral infarcts, ^ mRS >3 at 6 months. * at admission, # within 3 days after ictus, angiographically confirmed. Significant values are in **bold**.

Table S4. Univariate analysis of adverse events. The ventricular indices are dichotomized according to the AUC in the ROC analyses. In all ventricular indices, higher values correspond with longer fever and higher inflammatory parameters in serum and CSF.

**Parameter**	**Univariate analysis**		
	**Absent (Mean ±SD)**	**Present (Mean ±SD)**	**p-value**
Bifrontal ratio ≥ 0.337			
Age, years	50.99 (±12.70)	58.77 (±14.09)	**<0.0001**
ICP therapy, days	1.81 (±2.29)	1.71 (±2.65)	0.145
Fever, days	**4.61 (±4.10)**	**5.42 (±4.08)**	**0.007**
CRP at admission (mg/dl)	**0.9 (±1.84)**	**1.38 (±2.44)**	**0.001**
Mean 14-days CRP (mg/dl)	5.82 (±3.93)	6.27 (±4.20)	0.193
WBC at admission (x10^9/L)	13.42 (±4.94)	13.40(±4.67)	0.772
Mean 14-days WBC (x10^9/L)	12.16 (±3.81)	12.38 (±3.46)	0.262
CSF IL-6 at admission (pg/ml)	3620.47 (±6212.38)	4487.92 (±6958.39)	0.157
Mean 14-days CSF IL-6 (pg/ml)	**4789.11 (±6240.04)**	**5481.71 (±5151.54)**	**0.009**
Mechanical ventilation, days	6.81 (±6.12)	7.26 (±6.07)	0.209
Vasospasm on TCD, days	**2.76 (±3.47)**	**1.94 (±2.91)**	**0.003**
Bicaudate ratio ≥ 0.175			
Age, years	51.13 (±12.71)	60.64 (±13.81)	**<0.0001**
ICP therapy, days	1.87 (±2.50)	1.61 (±2.44)	0.134
Fever, days	**4.62 (±4.06)**	**5.68 (±4.10)**	**0.001**
CRP at admission (mg/dl)	**0.97 (±1.96)**	**1.39 (±2.44)**	**<0.0001**
Mean 14-days CRP (mg/dl)	**5.74 (±3.87)**	**6.59 (±4.36)**	**0.022**
WBC at admission (×10^9/L)	13.44(±4.52)	13.37 (±5.19)	0.409
Mean 14-days WBC (×10^9/L)	12.11 (±3.45)	12.57 (±3.80)	0.146
CSF IL-6 at admission (pg/ml)	3944.76 (±7247.45)	4386.61 (±5940.23)	0.110
Mean 14-days CSF IL-6 (pg/ml)	**4613.92 (±5775.74)**	**5861.78 (±5439.29)**	**<0.0001**
Mechanical ventilation, days	**6.63 (±6.10)**	**7.71 (±6.06)**	**0.009**
Vasospasm on TCD, days	**2.70 (±3.40)**	**1.79 (±2.82)**	**0.001**
Evans' index ≥ 0.281			

Age, years	52.35 (±13.07)	57.96 (±14.45)	**<0.0001**
ICP therapy days	1.95 (±2.49)	1.74 (±2.63)	0.093
Fever days	**4.74 (±4.07)**	**5.57 (±4.12)**	**0.008**
CRP at admission (mg/dl)	**1.00 (±2.02)**	**1.31 (±2.33)**	**0.002**
Mean 14-days CRP (mg/dl)	5.80 (±3.87)	6.36 (±4.25)	0.120
WBC at admission (x10⁹/L)	13.42 (±4.87)	13.38 (±4.69)	0.881
Mean 14-days WBC (x10⁹/L)	12.28 (±3.79)	12.30 (±3.41)	0.737
CSF IL-6 at admission (pg/ml)	3373.09 (±5745.11)	4856.04 (±7346.77)	0.134
Mean 14-days CSF IL-6 (pg/ml)	**4577.24 (±5900.42)**	**5730.27 (±5279.91)**	**0.001**
Mechanical ventilation, days	6.96 (±6.11)	7.35 (±6.05)	0.285
Vasospasm on TCD, days	2.60 (±3.38)	2.14 (±3.08)	0.082
Ventricular *ratio* ≥ 0.559			
Age, years	51.64 (±13.16)	60.56 (±13.65)	**<0.0001**
ICP therapy days	1.93 (±2.53)	1.67 (±2.43)	0.189
Fever days	**4.80 (±4.07)**	**5.61 (±4.12)**	**0.013**
CRP at admission (mg/dl)	**0.98 (±2.06)**	**1.43 (±2.32)**	**<0.0001**
Mean 14-days CRP (mg/dl)	**5.76 (±4.04)**	**6.58 (±4.01)**	**0.007**
WBC at admission (x10⁹/L)	13.45 (±4.53)	13.32 (±5.25)	0.367
Mean 14-days WBC (x10⁹/L)	12.18 (±3.52)	12.50 (±3.83)	0.342
CSF IL-6 at admission (pg/ml)	**4016.12 (±7220.72)**	**4218.09 (±5556.59)**	**0.020**
Mean 14-days CSF IL-6 (pg/ml)	**4555.10 (±5592.24)**	**6036.05 (±5585.13)**	**<0.0001**
Mechanical ventilation, days	6.91 (±6.13)	7.49 (±5.98)	0.129
Vasospasm on TCD, days	**2.59 (±3.38)**	**1.99 (±2.97)**	**0.021**
Huckmans index ≥ 5.480			
Age, years	50.59 (±12.74)	59.44 (±13.73)	**<0.0001**
ICP therapy days	**2.07 (±2.53)**	**1.62 (±2.53)**	**0.002**
Fever days	**4.71 (±4.13)**	**5.57 (±4.04)**	**0.003**
CRP at admission (mg/dl)	**0.89 (±1.83)**	**1.41 (±2.45)**	**<0.0001**
Mean 14-days CRP (mg/dl)	5.82 (±3.98)	6.36 (±4.12)	0.066
WBC at admission (x10⁹/L)	13.40 (±4.69)	13.44 (±4.89)	0.971
Mean 14-days WBC (x10⁹/L)	12.34 (±3.79)	12.26 (±3.44)	0.964
CSF IL-6 at admission (pg/ml)	4000.47 (±7411.59)	4274.74 (±5841.40)	0.186
Mean 14-days CSF IL-6 (pg/ml)	**4614.28 (±5915.41)**	**5620.68 (±5293.95)**	**0.001**
Mechanical ventilation, days	6.86 (±6.08)	7.49 (±6.07)	0.098
Vasospasm on TCD, days	**2.82 (±3.48)**	**1.91 (±2.92)**	**0.001**
3rd ventricle ratio ≥ 0.053			
Age, years	50.02 (±12.22)	59.24 (±14.09)	**<0.0001**
ICP therapy days	**2.15 (±2.67)**	**1.50 (±2.28)**	**0.001**
Fever days	**4.65 (±4.08)**	**5.54 (±4.10)**	**0.004**
CRP at admission (mg/dl)	**0.98 (±2.06)**	**1.32 (±2.30)**	**<0.0001**
Mean 14-days CRP (mg/dl)	**5.62 (±3.87)**	**6.37 (±4.16)**	**0.020**
WBC at admission (x10⁹/L)	13.40 (±4.70)	13.30 (±4.79)	0.914
Mean 14-days WBC (x10⁹/L)	12.17 (±3.69)	12.32 (±3.47)	0.223
CSF IL-6 at admission (pg/ml)	4574.69 (±8471.86)	4059.89 (±5564.72)	0.397
Mean 14-days CSF IL-6 (pg/ml)	**4270.01 (±4987.71)**	**5865.82 (±6117.89)**	**<0.0001**
Mechanical ventilation, days	6.85 (±5.97)	7.42 (±6.20)	0.204
Vasospasm on TCD, days	**2.78 (±3.47)**	**2.04 (±3.03)**	**0.009**

Abbreviations: AUC= area under the curve, ROC= receiver operating characteristics, ICP= intracranial pressure, CRP= C-reactive protein, CSF= cerebrospinal fluid, IL-6= interleukin-6, WBC= white blood cells count, TCD= transcranial Doppler sonography. Significant findings are in **bold**.

Table S5. Multivariate analyses of the predictors of the secondary study endpoints using binary logistic regression with obligatory inclusion of major confounders (patients' age and WFNS, Fisher and SEBES grades at admission) enhanced by the ventricle indices included in the stepwise backward regression model (only the last steps are shown here). For the multivariate analysis of the predictors of mean 14-days CSF IL-6, bacterial meningitis was included as an additional confounder to the prediction model. The continuous endpoints were dichotomized according to the common reference values or the mean/median values within the analyzed cohort. Only the last step of each multivariate analysis is shown.

**Decompressive craniectomy (DC)**				
Age >55 years	0.77	0.51	1.16	0.211
**WFNS grade 4–5**	**2.90**	**1.92**	**4.36**	**<0.0001**
**SEBES 3–4**	**2.17**	**1.44**	**3.25**	**<0.0001**
**Fisher grade 3–4**	**6.25**	**2.14**	**18.21**	**0.001**
Bifrontal ratio>0.337	0.69	0.45	1.06	0.092
**Ventricular ratio >0.559**	**0.43**	**0.26**	**0.70**	**0.001**
Third ventricular ratio >0.053	0.66	0.42	1.04	0.076
**Any ICP increase requiring conservative and/or surgical (DC) treatment**				
**Age >55 years**	**0.64**	**0.45**	**0.93**	**0.018**
**WFNS grade 4–5**	**3.16**	**2.21**	**4.51**	**<0.0001**
**SEBES 3–4**	**2.22**	**1.57**	**3.15**	**<0.0001**
**Fisher grade 3–4**	**2.74**	**1.44**	**5.22**	**0.002**
**Third ventricular ratio >0.053**	**0.65**	**0.46**	**0.93**	**0.017**
**CRP at admission ≥ 0.5 mg/dL**				
Age >55 years	098	0.68	1.40	0.910
WFNS grade 4–5	1.11	0.78	1.57	0.563
SEBES 3–4	1.15	0,.81	1.63	0.436
Fisher grade 3–4	1.20	0.69	2.08	0.514
**Evans ratio >0.2813**	**1.55**	**1.11**	**2.18**	**0.011**
**Ventricular ratio >0.559**	**1.49**	**1.04**	**2.13**	**0.031**
**Aneurysm rebleeding**				
Age >55 years	1.49	0.78	2.87	0.228
**WFNS grade 4–5**	**2.07**	**1.04**	**4.14**	**0.040**
SEBES 3–4	1.28	0.66	2.49	0.460
Fisher grade 3–4	1.46	0.32	6.61	0.625
**TCD Vasospasm**				
**Age >55 years**	**0.52**	**0.36**	**0.75**	**<0.0001**
WFNS grade 4–5	1.21	0.84	1.76	0.306
SEBES 3–4	**2.24**	**1.56**	**3.21**	**<0.0001**
Fisher grade 3–4	1.56	0.88	2.79	0.131
**Fever for >5 days**				
Age >55 years	0.71	0.49	1.01	0.058
WFNS grade 4–5	**1.83**	**1.29**	**2.59**	**0.001**
SEBES 3–4	1.12	0.79	1.60	0. 519
Fisher grade 3–4	**3.72**	**1.93**	**7.19**	**<0.0001**
Evans' index >0.281	1.55	1.10	2.18	0.012
**Systemic infections**				
Age >55 years	1.14	0.80	1.62	0.473
WFNS grade 4–5	**2.00**	**1.41**	**2.83**	**<0.0001**
SEBES 3–4	0.83	0.58	1.18	0.298
Fisher grade 3–4	**2.87**	**1.45**	**5.65**	**0.002**
**Acute renal failure**				
Age >55 years	0.73	0.15	3.56	0.696
WFNS grade 4–5	2.52	0.49	13.04	0.270
SEBES 3–4	0.37	0.06	2.10	0.260
Fisher grade 3–4	9,908,780.60	-		0.997

Bicaudate ratio >0.175	7.35	0.83	64.81	0.073
**High mean 14–days CSF IL–6 (>3300 mg/dL)**				
Age >55 years	**2.10 (1.26–3.52)**			**0.005**
WFNS grade 4–5	1.62 (0.99–2.65)			0.057
SEBES 3–4	1.05 (0.87–1.27)			0.618
Fisher grade 3–4	1.14 (0.37–3.46)			0.823
Bacterial meningitis	**2.80 (1.67–4.69)**			**<0.0001**
3rd ventricle ratio >0.053	**1.69 (1.01–2.82)**			**0.047**

Abbreviations: aOR= adjusted odds ratio, CI= confidence interval, ROC= receiver operating characteristic, SEBES= subarachnoid hemorrhage early brain edema score, WFNS= world federation of neurosurgical societies, TCD= transcranial Doppler sonography, CSF= cerebrospinal fluid, IL-6= Interleukin-6, ICP= intracranial pressure. Significant findings are in **bold**.

Disclaimer/Publisher's Note: The statements, opinions and data contained in all publications are solely those of the individual author(s) and contributor(s) and not of MDPI and/or the editor(s). MDPI and/or the editor(s) disclaim responsibility for any injury to people or property resulting from any ideas, methods, instructions or products referred to in the content.
